# Directed Evolution of *Dunaliella salina Ds-26-16* and Salt-Tolerant Response in *Escherichia coli*

**DOI:** 10.3390/ijms17111813

**Published:** 2016-10-29

**Authors:** Yuan Guo, Yanping Dong, Xiao Hong, Xiaonan Pang, Defu Chen, Xiwen Chen

**Affiliations:** 1 Department of Biochemistry and Molecular Biology, College of Life Sciences, Nankai University, No. 94 Weijin Rd., Tianjin 300071, China; guoyuan09143121@126.com (Y.G.); ypdong@163.com (Y.D.); 2 Department of Genetics and Cell Biology, College of Life Sciences, Nankai University, No. 94 Weijin Rd., Tianjin 300071, China; jenelolen@outlook.com (X.H.); fightingnk@163.com (X.P.)

**Keywords:** *Ds-26-16*, directed evolution, salt-tolerant response, abiotic stress, *Escherichia coli*

## Abstract

Identification and evolution of salt tolerant genes are crucial steps in developing salt tolerant crops or microorganisms using biotechnology. *Ds-26-16*, a salt tolerant gene that was isolated from *Dunaliella salina*, encodes a transcription factor that can confer salt tolerance to a number of organisms including *Escherichia coli* (*E*. *coli*), *Haematococcus pluvialis* and tobacco. To further improve its salt tolerance, a random mutagenesis library was constructed using deoxyinosine triphosphate-mediated error-prone PCR technology, and then screened using an *E*. *coli* expression system that is based on its broad-spectrum salt tolerance. Seven variants with enhanced salt tolerance were obtained. Variant EP-5 that contained mutation S32P showed the most improvement with the *E*. *coli* transformant enduring salt concentrations up to 1.54 M, in comparison with 1.03 M for the wild type gene. Besides, *Ds-26-16* and *EP-5* also conferred *E*. *coli* transformant tolerance to freezing, cold, heat, Cu^2+^ and alkaline. Homology modeling revealed that mutation S32P in EP-5 caused the conformational change of N- and C-terminal α-helixes. Expression of *Ds-26-16* and *EP-5* maintained normal cellular morphology, increased the intracellular antioxidant enzymatic activity, reduced malondialdehyde content, and stimulated Nitric Oxide synthesis, thus enhancing salt tolerance to *E*. *coli* transformants.

## 1. Introduction

Soil salinization has become a global issue associated with human production activities. Under high salt conditions, the metabolic activities of plants, such as photosynthesis, respiration, and ion transport, are greatly affected, resulting in growth retardation, production decline and even death [1]. In industrial fermentation processes, it is often necessary to add high concentrations of sugar or minerals to the filler. The microorganism is easily encountered with salt and hyperosmotic stress, and the growth and productivity are reduced [2]. Therefore, how to improve the salt tolerance of organisms has become a critical problem for scientists. Identifying key genes that confer salt tolerance and elucidating their mechanisms are crucial steps in salt-tolerance breeding using biotechnology.

Currently, a large number of genes related to salt stress have been identified by positional cloning, homologous cloning, T-DNA insertion, cDNA microarrays, high-throughput gene expression and whole-genome sequencing technology [1,3]. Proteins encoded by these genes include transcription factors that sense and regulate the expression of stress response, such as NAM, ATAF and CUC (NAC) and a kind of DNA binding protein which can combine with W box specifically (WRKY) transcription factors; protein kinases that act as a molecular switch to regulate salt stress response signals; proteins involved in membrane transport and water channel, such as Na^+^/H^+^ antiporter, osmolyte synthase; proteins associated with photosynthetic pathway and other carbon metabolic pathways; oxidative stress related proteins, and chaperones such as heat shock protein [1,3,4,5]. These genes have been extensively applied in genetic transformation [5]. However, most of them were from glycophytes, and the salt tolerance was restricted by the evolution, while identifying salt-tolerant genes from special habitat resources was largely underestimated. Therefore, the salt-tolerance of these genes still needs to be further enhanced.

In theory, protein in nature has great potential for evolution. Rational and irrational designs are two common strategies in protein engineering. Rational design is usually based on information about the structure–function relationship, and is then aimed at important sites for engineering, a strategy which is quite effective [6]. However, due to the necessity of understanding the structure and function of proteins, application of the strategy is quite restricted. Directed evolution, which consists of DNA random mutation and screening for variants with specific phenotypes, is suitable for modification of salt-tolerant genes with little information on the structure–function activity. For instance, error-prone PCR was used to modify *HvHAK1* encoding a K^+^ transporter in barley, the evolutionary gene could confer *Saccharomyces cerevisiae* transformants tolerance to 750 mM NaCl [7]. A Na^+^/H^+^ antiporter gene *SseNHX1*, which is a chimeric gene of *SsNHX1* and *SeNHX1* (from halophytes *Suaeda salsa* and *Salicornia europaea*, respectively), was obtained by DNA family shuffling. The gene enhanced salt tolerance by 146% and 122% more than the template, respectively [8].

Recently, directed evolution has begun to be used in engineering non-catalytic proteins, such as transcription factors. Evolution of transcription factors is supposed to reprogram transcription of a large number of genes and elicit cellular phenotypes important for technological applications. This approach has been successfully applied in prokaryotic cells [9,10,11], and in eukaryotic yeast [12,13]. For instance, by engineering global transcription factor cAMP receptor protein, the evolutionary gene rendered *Escherichia coli* (*E*. *coli*) transformant tolerating the highest salt concentration from 0.9 to 1.1 M [11]. Mutagenesis of the transcription factor Spt15p led to dominant mutations that conferred higher tolerance and more efficient glucose conversion to ethanol [12]. Evolution of transcription factor could also enhance the transcription activity and thus improve drought tolerance of the transgenic plants [14,15,16]. Transcription factors are generally located in the middle of signaling pathway, and play a connecting role—perceiving upstream stress signals and specifically regulating expression of downstream function genes [17]. Meanwhile, transcription factors are involved in integration and crosstalk among multiple signaling pathways [2,11,18]. Therefore, evolution of a single transcription factor is expected to alter expression of multiple genes simultaneously, which may not be achieved by sequential multigene modifications.

We have previously isolated a novel salt-tolerant gene *Ds-26-16* from a 4 M NaCl-induced *Dunaliella salina* cDNA library. The gene contained a 159 bp open reading frame, and could confer salt tolerance to several organisms, including *Haematococcus pluvialis*, tobacco and *E*. *coli*. Subcellular localization showed that Ds-26-16 localizes to nucleus. Bioinformatics revealed that Ds-26-16 has homology with zinc finger protein, DNA binding proteins. Therefore, it is considered as a broad-spectrum trans-regulatory gene with salt-tolerance [19]. Isobaric tags for relative and absolute quantitation (iTRAQ) analysis showed that Ds-26-16 upregulated five transcription factors, and adjusted the osmotic balance, energy metabolism and oxidation protection to confer salt tolerance to *E. coli* [20]. To further enhance the salt tolerance, we constructed a random mutation library using deoxyinosine triphosphate (dITP)-mediated Error-prone PCR (EP-PCR), a random mutagenesis technique for introducing amino acid changes into proteins, and screened the variants using the *E. coli* expression system. Our data suggested that mutations in transcript factor Ds-26-16 could effectively enhance the salt tolerance to *E*. *coli*. Expression of *Ds-26-16* and *EP-5* maintained the cellular morphology, increased the intracellular antioxidant capability, and stimulated Nitric Oxide (NO) synthesis to confer salt tolerance to *E*. *coli*. This study provided basis for further utilization of Ds-26-16 and the evolutionary gene in salt tolerance breeding.

## 2. Results

### 2.1. Construction of Random Mutant Library and Screening for Salt-Tolerance Variants

To improve the mutation frequency, two rounds of a deoxyribonucleic analogue dITP-mediated error-prone PCR were performed according to the conditions listed in Table S1. After collecting the PCR product and inserting it into *E. coli*, a random mutant library of *Ds-26-16* with a capacity of 1 × 10^5^ CFU/μg was obtained. PCR analysis revealed that 80% of cells carried *Ds-26-16*.

After screening, 7 variants that were able to grow above 1.54 M NaCl were obtained. Sequencing revealed that all the 7 variants had a single nucleotide substitution that resulted in a single amino acid residue substitution. These variants are EP-3 (F23L), EP-5 (S32P), EP-136 (W2G), EP-171 (P24T), EP-176 (N49D), EP-179 (S46G) and EP-183 (with addition of 16 more amino acid residues because of the mutation at the terminator). Of them, EP-5 showed the highest and most consistent salt tolerance and was used in subsequent study, while other variants were not characterized any more here owing to the inconsistent salt tolerance.

### 2.2. EP-5 Significantly Improved the Salt Tolerance to E. coli Transformant

**Figure 1:**
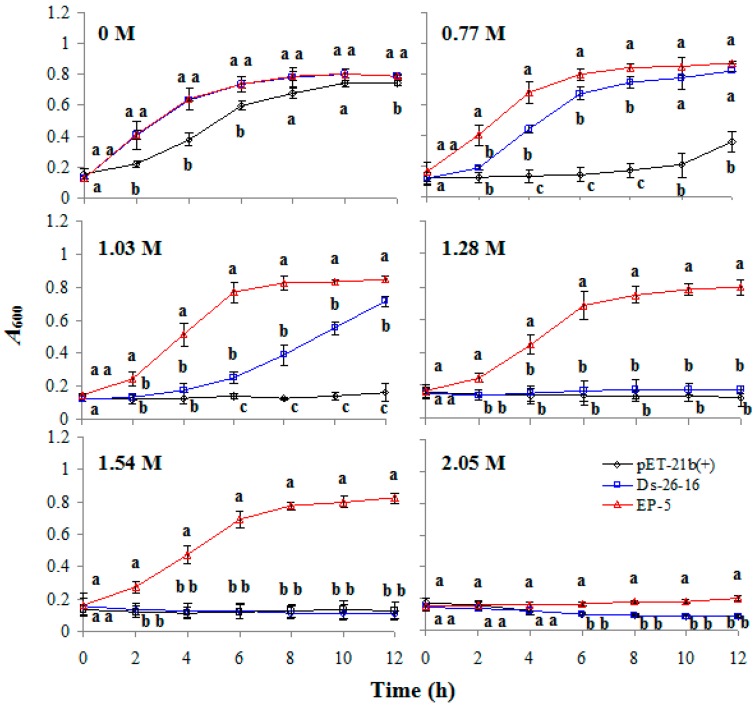
The salt tolerance of EP-5 transformant. The growth status (*A*_600_) of pET-21b(+), Ds-26-16 and EP-5 under different NaCl concentrations. a, b or c stands for the significant differences between the strains under the same concentration at the same growth time (*p* < 0.05).

In order to quantify the salt tolerance of EP-5 to *E. coli*, the growth of pET-21b(+), Ds-26-16 and EP-5 under different concentrations of NaCl was determined. Under control conditions (0 M NaCl), the *A*_600_ value of Ds-26-16 and EP-5 had no significant difference, but was higher than that of pET-21b(+). With the increase of NaCl concentration, the differences in the growth among these strains became more obvious. Under 0.77 and 1.03 M NaCl, the growth of pET-21b(+) was severely inhibited, while EP-5 and Ds-26-16 grew well, with EP-5 entering logarithmic phase earlier than Ds-26-16. Under 1.28 and 1.54 M NaCl, EP-5 grew rapidly but Ds-26-16 severely inhibited the growth. Only under 2.05 M NaCl, the growth of EP-5 began to be inhibited (Figure 1). These results indicated that EP-5 could tolerate NaCl concentrations up to 1.54 M, in comparison with 1.03 M for the wild type gene.

### 2.3. EP-5 Conferred Other Abiotic Tolerances to E. coli Transformants

**Figure 2:**
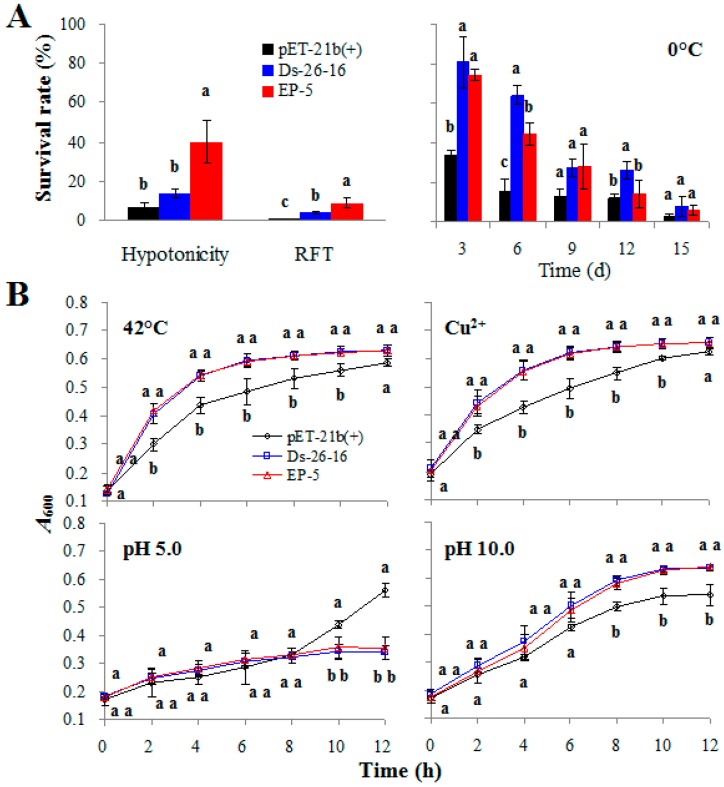
Characteristics of EP-5 to abiotic tolerance other than salt tolerance. (**A**) The survival rate of pET-21b(+), Ds-26-16 and EP-5 after hypotonicity and repeated freezing treatment (RFT); (**B**) The growth status of pET-21b(+), Ds-26-16 and EP-5 under various abiotic stresses, respectively. a, b or c stands for the significant differences (*p* < 0.05) between the strains under the same stress at the same growth time (*p* < 0.05).

Various abiotic stresses usually have some mutual regulatory mechanisms or signaling pathways, and a salt tolerant transcription factor may also be involved in other abiotic stress tolerance [21]. To this end, the tolerance of *EP-5* and *Ds-26-16* to other abiotic stress was also analyzed in *E. coli* transformants. Compared with the control strain pET-21b(+), Ds-26-16 strain had a higher survival rate after repeated freezing treatment (RFT) and 0 °C treatment (Figure 2A); had a higher growth rate under conditions of 42 °C, 10 mg/L Cu^2+^ and pH 10.0 (Figure 2B); showed no significant difference after hypotonic treatment (Figure 2A); but decreased the growth rate in acidic conditions (Figure 2B). Therefore, in addition to salt tolerance, *Ds-26-16* also conferred a stronger ability in resisting repeated freezing, cold, heat, Cu^2+^, and alkaline, but not hypotonicity and acid to *E. coli*.

Compared with Ds-26-16, EP-5 strain had a higher growth rate after freezing and hypotonicity treatment (Figure 2A); showed no difference under conditions of 42 °C, 10 mg/L Cu^2+^, pH 5.0 or pH 10.0 (Figure 2B); but decreased the survival rate after 0 °C treatment (Figure 2A). These results showed that, besides salt tolerance, EP-5 also improved its tolerance to freezing and hypotonicity, decreased its tolerance to cold, without changing the tolerance to heat, Cu^2+^, acid and alkaline.

### 2.4. S32P Mutation Changed the Relationship of Two α-Helixes on N- and C-Terminal in EP-5

**Figure 3:**
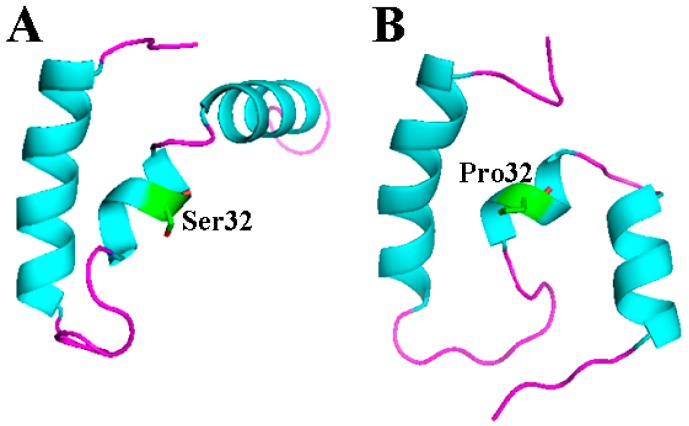
The comparison of the homology structure between EP-5 and Ds-26-16. The amino acid sequences of EP-5 and Ds-26-16 were submitted to Phyre2 server, respectively, for homology modeling in the intensive mode, which is based on a profile–profile alignment algorithm. (**A**) Ds-26-16; (**B**) EP-5. The blue parts stands for α-helixes, the green part is residue 32, and the purple line is loop.

The function of a protein is usually related to the structure. To analyze the structural changes resulting from the mutation S32P, homology modeling of EP-5 and Ds-26-16 was conducted. Sixteen templates were obtained, 12 of which showed identities higher than 46%, though all had a confidence lower than 30% (Table S2). The resulting modeling revealed that Ds-26-16 was composed of 3 α-helices. Among these helices, two bigger ones were located at the N- and C-terminal of the protein, respectively, and were perpendicular to each other. The other α-helix, which connects the two bigger helixes, contains residue Ser32 (Figure 3A). When Ser32 was mutated into Pro32, the small α-helix twisted and transformed the perpendicular orientation of the two bigger α-helices into a parallel orientation (Figure 3B). However, whether this change is directly related to the improvement of EP-5 to salt tolerance needs to be further verified.

### 2.5. EP-5 Maintained the Normal Cell Morphology of E. coli Transformant

Osmotic stress significantly influences the cell morphology of bacteria [22]. To investigate the effect of Ds-26-16 and EP-5 on cell morphology, scanning electron microscope (SEM) was employed to examine the *E*. *coli* transformants under NaCl condition (Table 1, Figure S1). In the absence of NaCl (0 M), all strains had a typical rod shape and their average cell length was similar (2.23–2.37 μm). Under 0.51 M NaCl, pET-21b(+) became extremely elongated (31.25 μm), while EP-5 and Ds-26-16 was still 2.36–2.70 μm. Under 1.03 M NaCl, half of pET-21b(+) cells were still filamentous and the other half were close to normal shape, while both EP-5 and Ds-26-16 elongated to some degree. Under 1.54 M NaCl, pET-21b(+) showed no filamentous but cells shrank and its average length was 1.81 μm, while EP-5 and Ds-26-16 still became elongated, and EP-5 (3.74 μm) elongated more obviously than Ds-26-16 (3.06 μm). In short, with NaCl concentration increasing, the cell length of EP-5 and Ds-26-16 were relatively stable, but pET-21b(+) became filamentous at lower NaCl concentrations and compressed at higher NaCl concentrations.

In addition, we also observed the percentage of broken cells after fixation, dehydration and drying procedures. pET-21b(+) was easily damaged and the broken cells accounted for about 55% (Figure S1), while Ds-26-16 and EP-5 were relatively tolerant to this external damage. This result is coincident with the results of repeated freezing and hypotonicity tolerance as shown in Figure 2A.

### 2.6. EP-5 Improved the Antioxidant Capacity of E. coli Transformant

**Figure 4:**
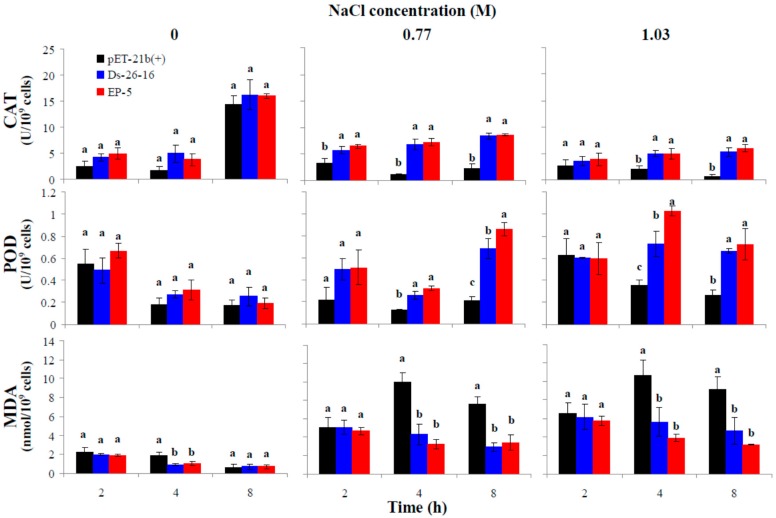
Antioxidant ability of *E*. *coli* transformants expressing Ds-26-16 or EP-5 under different NaCl stress levels. Catalase (CAT) and peroxidase (POD) activities and malondialdehyde (MDA) content were determined after pET-21b(+), Ds-26-16 and EP-5 cultured under NaCl stress at different times. a, b or c stands for the significant differences (*p* < 0.05) between the strains under the same salt concentration at the same growth time.

High concentration of salt in environment leads to the accumulation of reactive oxygen species (ROS), and therefore enhancement of intracellular antioxidant capacity is closely related to salt tolerance. To investigate the effects of *EP-5* and *Ds-26-16* on the intracellular antioxidant ability, catalase (CAT) and peroxidase (POD) activities of *E. coli* transformants under NaCl were determined (Figure 4). Overall, CAT activity increased with time, while POD activity showed no obvious pattern. Under 0 M NaCl, three strains showed no difference in two enzymatic activities. Under 0.77 M NaCl, CAT and POD activities of Ds-26-16 and EP-5 was significantly higher than that of pET-21b(+). At 8 h, CAT activities of Ds-26-16 and EP-5 were 3.70-fold and 3.78-fold of that of the control, respectively, while POD activity of Ds-26-16 and EP-5 were 3.30-fold and 4.15-fold higher than that of the control. POD activity of EP-5 was significantly higher than that of the Ds-26-16 (1.26-fold). Overall, CAT activity at 1.03 M was lower than that of 0.77 M, but POD activity at 1.03 M was obviously higher than that of 0.77 M. Under 1.03 M NaCl stress, the activities of Ds-26-16 and EP-5 were still significantly higher than that of the control. At 8 h, CAT activities of Ds-26-16 and EP-5 were 9.56-fold and 10.82-fold higher than that of the control, respectively; and POD activities of Ds-26-16 and EP-5 were 2.53-fold and 2.75-fold higher than that of the control; also, POD activity of EP-5 was significantly higher than that of the Ds-26-16 (1.41-fold). These results indicated that improvement of EP-5 to salt tolerance in *E*. *coli* transformants is related to the increase of the activities of CAT and POD under salt stress.

Accumulation of ROS would induce the peroxidation of membrane lipid, and malondialdehyde (MDA) is a peroxidation product. To investigate the effect of *EP-5* and *Ds-26-16* on membrane damage, MDA content of *E*. *coli* transformants under salt stress was determined (Figure 4). Under 0 M NaCl, MDA content decreased with time. MDA contents of Ds-26-16 and EP-5 were lower than that of the control, but only at 4 h did they show significant difference. At 4 h, MDA contents of Ds-26-16 and EP-5 were 0.50- and 0.55-fold of that of the control, respectively. Under 0.77 M NaCl, MDA content of the control strain increased first and then decreased with time, while that of Ds-26-16 and EP-5 changed without an obvious pattern. At 8 h, MDA contents of Ds-26-16 and EP-5 were 0.39- and 0.45-fold lower than that of the control, respectively. MDA content changed similarly at 1.03 M to that at 0.77 M. MDA content of EP-5 at each NaCl concentration was slightly lower than that of Ds-26-16. These results indicated that improvement of EP-5 to salt tolerance in *E. coli* transformants is related to the decrease in membrane damage.

### 2.7. EP-5 Stimulated NO Synthesis in E. coli Transformant

**Figure 5:**
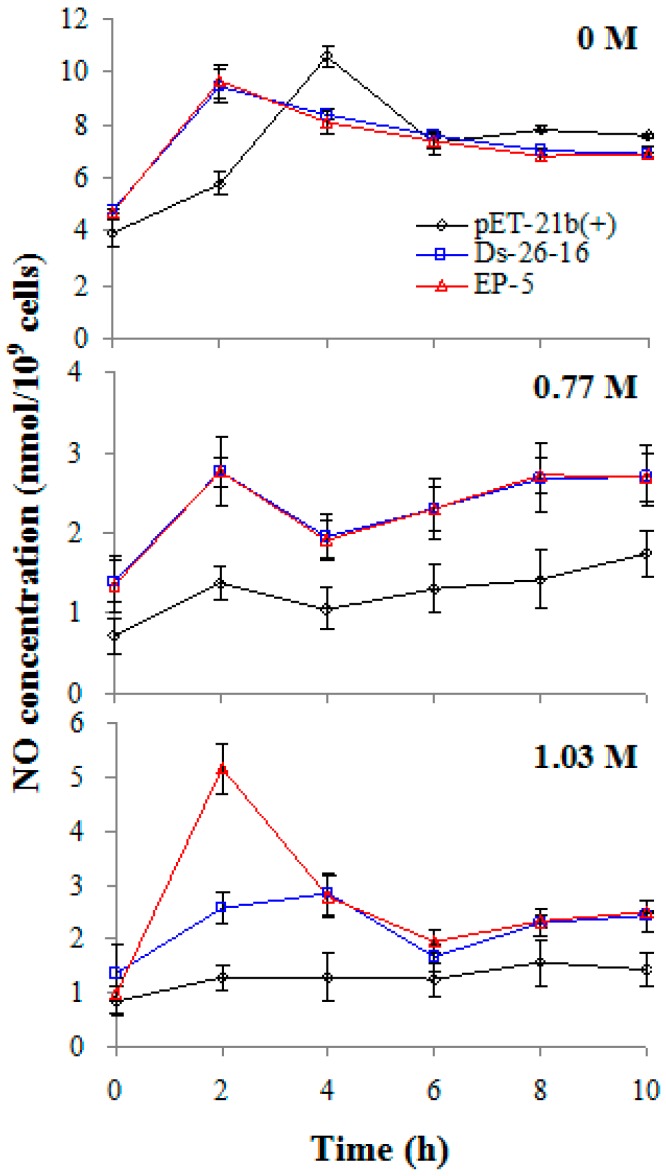
Nitric oxide content of *E*. *coli* transformants expressing Ds-26-16 or EP-5 under different NaCl stress. Nitric oxide was determined after pET-21b(+), Ds-26-16 and EP-5 were cultured under NaCl stress at different times.

NO is a signaling that involved in salt and osmotic stress response [23]. To investigate the effect of *EP-5* and *Ds-26-16* on endogenous NO synthesis, NO content of *E. coli* transformants under salt stress was determined (Figure 5). NO content increased first and then decreased with the time, and its peak emerged in the early stage of logarithmic phase, suggesting the close relationship of NO synthesis with cell growth. NO content cultured under 0 M NaCl was higher than that under salt stress (0.77 and 1.03 M), indicating that salt stress reduced intracellular NO content. Under 0 M NaCl, NO content peaks of EP-5 and Ds-26-16 appeared at the same time (2 h), earlier than that of pET-21b(+) (4 h). Under 0.77 M NaCl, two strains still appeared to peak at the same time, but NO content of EP-5 was much higher than that of Ds-26-16. Under 1.03 M conditions, the peak of EP-5 appeared at 2 h, earlier than that of Ds-26-16 (4 h), and the peak value was 1.82-fold higher than that of Ds-26-16; while NO content of pET-21b(+) remained at a low level throughout. These results indicated that improvement of EP-5’s salt tolerance in *E. coli* transformant is related to the increase in endogenous NO synthesis.

## 3. Discussion

Protein engineering is composed of rational and irrational design. Rational design is applicable to proteins with clear structural knowledge, and irrational design (directed evolution) is suitable for proteins whose structures have not been studied in depth. We previously screened *Ds-26-16*, a salt tolerance gene from *Dunaliella salina*. Nucleotide BLAST (The Basic Local Alignment Search Tool) analysis revealed that *Ds-26-16* had no homolog in GenBank. Therefore, in this study, we utilized directed evolution to modify *Ds-26-16*. Construction of a mutation library with high abundance and appropriate mutation rate is one of the key determinants of evolution [24]. If the mutation rate is too low, it would lower the frequency to screen out variants. On the contrary, if the mutation rate is too high, large numbers of deleterious mutations would hide the beneficial ones [24]. Due to the short open reading frame (159 bp), it is not easy to create enough random mutations using traditional error prone PCR, such as increasing concentrations of Mg^2+^ and Mn^2+^. Therefore, dITP, a deoxyribonucleic analogue that could pair with C, T or A [25], was supplied here to mediate the reaction. The effect was demonstrated in our study as seven variants were screened from the library. However, we did not get more variants, which may be attributed to the following reasons. First, the mutation library we constructed is not big enough (a capacity of 1 × 10^5^ CFU/μg). We tried to increase the capacity. However, as the template gene is too short, it is not easy to collect enough mutated DNA for library construction. Second, Ds-26-16, as a transcription factor (different from a catalytic protein), might be more conservative and the evolution potential might be limited. Third, salt stress response is a complex physiological process involving multiple genes and signaling pathways. Evaluation of salt tolerance is not as distinct in phenotypes controlled by a single gene. It is easily influenced by biological and environmental adaptabilities. Therefore, exploring efficient mutagenesis strategies and screening methods constitutes a future direction of this work.

Transcription factor, through interactions with *cis*-regulatory sequences, can regulate the expression of a number of genes. Modifications of a transcription factor can alter its DNA binding properties, interaction properties with its chaperone proteins or transcription regulation elements [26], which lead to reprogramming transcription of a large number of genes and changes in cellular phenotypes that are valuable for technological applications. Evolution of a transcription factor was also reported to enhance the transcription activity by deleting the repressor region and forming a constitutive active state [15,16]. As Ds-26-16 was a structurally unknown transcription factor, it is difficult to explain how the evolution enhanced the salt tolerance capability of *E. coli* transformants. However, the screened variants enhanced the salt tolerance than the wild type, suggesting that enhancing the transcription activity may be the main reason to increase the salt tolerance. Furthermore, most (6/7) of the screened variants contained substitution at middle (3/7) or C-terminal (3/7), also suggesting that the binding domain possibly resided at the C-terminal of the protein. This also coincided with the finding that Ds-26-16 identified highly with several DNA-binding proteins at the C-terminal (Table S2). From the spatial structure (though it is not sufficiently reliable), mutation S32P in EP-5 twisted the α-helixes in the collecting region and may enhance the binding capability with regulatory sequences. Further research is needed to clarify how the evolution enhanced the salt tolerance of Ds-26-16 as a transcription factor.

Cell morphology in microorganisms is a visible indicator of their adaptation to stress conditions. Under high NaCl conditions, bacteria form elongated filamentous cells. Such cells have intact membranes and could revert to normal morphology upon removal of stress. Therefore, cell filamentation could be considered as a means of bacteria adaptation in unfavorable conditions [22]. This has been reported in a number of organisms including *Gluconacetobacter diazotrophicus* PAL5 [27], *Salmonella* [22], and also *E*. *coli* [28]. In this study, the observation of elongated cells in *E. coli* transformants suggested that cell elongation is also a strategy of *E*. *coli* adapting to salt stress. However, the situation was only occurred in a lower salt concentration. With the salt concentration increasing, *E*. *coli* cells would partly or totally lose the capability and become contracted. pET-21b(+) cells elongated at lower salt concentrations (0.51 M NaCl) than that of EP-5 and Ds-26-16 (1.03 M), suggesting a stronger tolerance of EP-5 and Ds-26-16 to salt stress than pET-21b(+). The cell length of EP-5 was bigger than Ds-26-16 in a normal morphological range under salt stress, suggesting the stronger tolerance of EP-5 than Ds-26-16, which supports Zhang’s observation that *E*. *coli* transformants became elongated to some degree when its NaCl tolerance improved [11]. However, it is still unclear how the gene caused cell filamentation, and the causal linkage between the gene and salt tolerance needs further study.

Various biotic or abiotic stresses would result in accumulation of reactive oxygen species (ROS). Organisms have developed several defense mechanisms including enzymatic and non-enzymatic antioxidant systems. Previous research demonstrated that salt tolerance is closely related to the efficiency of antioxidant systems [29]. In this study, expression of *EP-5* resulted in higher POD and CAT activities and lower MDA content in *E*. *coli* transformants under salt stress than that of Ds-26-16, suggesting that improving the antioxidant systems is one of the mechanisms through which *EP-5* resists salt stress in *E*. *coli*. NO can counteract part of ROS mediated toxicity and alleviate the salt stress [30]. NO is also involved in the salt response signaling pathway by activating related kinases [23] and inducing the expression of ion transporter genes [31]. Our previous study suggested that enhanced nitric oxide synthesis via lower intercellular pH was one of the mechanisms by which Ds-26-16 confers salt tolerance to *E*. *coli* [20]. In the study, *EP-5* significantly stimulated NO synthesis under salt stress and the growth of *E*. *coli* transformants is closely related to endogenous NO content, confirming the close relationship between endogenous nitric oxide synthesis and salt stress.

## 4. Materials and Methods

### 4.1. Plasmid, Chemical Reagents and Strains

The wild type *Ds-26-16* was derived from our previous work [19]. p21-ORF is the plasmid that inserted the open reading frame of *Ds-26-16* (159 bp) into the *Sal*I/*Hin*dIII site of pET-21b(+). dITP were obtained from Thermo Fisher Scientific. Inc. (Waltham, MA, USA). *rTaq* DNA polymerase were obtained from Dalian TaKaRa Biotech. Co., Ltd. (Dalian, China). NO detection kit was provided by Beyotime Biotechnology Inc. (Shanghai, China). *E*. *coli* DH5α-FT and BL21(DE3) were kept in our laboratory.

To simplify the description, LB medium containing a final concentration of 1 mM Isopropyl-*β*-d-thiogalactopyranside (IPTG) and 0.1 mg/mL Amp was named as LBA. 2× TY medium containing a final concentration of 1 mM IPTG and 0.1 mg/mL Amp was named as TYA. BL21(DE3) containing pET-21b(+) plasmid or p21-ORF plasmid were named as pET-21b(+) strain or Ds-26-16 strain, respectively. BL21(DE3) containing pET-21b(+) that carried the evolved gene of Ds-26-16 was named after its evolved gene.

### 4.2. Construction of Ds-26-16 Random Mutation Library

Two rounds of dITP, a deoxyribonucleic analogue-mediated random mutagenesis PCR (Table S1) was used for library construction. The first round PCR was conducted using plasmid p21-ORF as template, pET-Upstream/Seq-rew (Table S3) as primer pairs and 40 cycles for the reaction. The second round was conducted using the first round of PCR product as template, pET-T7/T7-Ter2 as primers and 32 cycles for the reaction. After collecting the products, the fragment was then inserted to the *Sal*I and *Hin*dIII site of pET-21b(+). The resulting plasmids were then transformed into competent cells of *E. coli*. The random mutation library was then evaluated with primer pairs of 159-EP-F/159-EP-R.

### 4.3. Screening for Ds-26-16 Variant

The bacteria suspension from the random mutation library was inoculated at 1/10 (*v*/*v*) quantity sequentially to liquid LBA medium containing NaCl from 0, 0.77, 1.03, 1.28, 1.54 to 2.05 M, respectively. To enrich the salt-tolerant strains, cultures at each salt gradient were kept for 12 h. As Ds-26-16 strain did not tolerate 1.54 M NaCl, the bacteria suspension that enriched in 2.05 M NaCl medium was then inoculated on solid LB medium containing 0.1 mg/mL Amp. After overnight incubation at 37 °C, the single colony was picked and then inoculated in LBA medium with progressively increased salt concentration for the second round of screening. The strains that grew faster than Ds-26-16 were collected under each salt gradient. The variant strains were identified using PCR and then sequenced.

### 4.4. Stress Tolerance Analysis

Salt tolerance analysis: strains (1.5 × 10^8^ cells/mL) were inoculated in TYA medium containing NaCl at 0, 0.77, 1.03, 1.28, 1.54 or 2.05 M, respectively, and then cultured on a shaker at 37 °C. The *A*_600_ value was measured every 2 h. The salt tolerance was evaluated according to the growth curve.

Tolerance to hypotension/freezing-thawing/low temperature: strains (1.5 × 10^8^ cells/mL) were inoculated in 2× TY medium containing 0.1 mg/mL Amp, and then cultured on a shaker at 37 °C. When *A*_600_ reached 0.4, IPTG (isopropyl *β*-d-1-thiogalactopyranoside) with a final concentration of 1 mM was added to the medium and continued to culture for 3 h. The cultures were diluted by 1:10^5^ (*v*/*v*) and then subjected to hypotonic shock (centrifuged at 5000 r/min for 5 min, then suspended in equal volume of distilled water and kept for 4 h), repeated freezing treatment (RFT, freeze-thaw for 3 times at −20/37 °C) or low-temperature treatment (kept at 0 °C for 0, 3, 6, 9, 12, and 15 days). Then, 0.1 mL cultures were inoculated on LB solid medium (containing 0.1 mg/mL Amp) at 37 °C overnight. The number of colonies was counted and the survival rate was calculated. The tolerance to hypotension/repeated freezing/low temperature was evaluated according to the survival rate.

Tolerance to high temperature: strains (1.5 × 10^8^ cells/mL) was inoculated in TYA medium on a shaker at 42 °C. The *A*_600_ value was measured every 2 h. The tolerance to high temperatures was evaluated according to the growth curve.

Tolerance to Cu^2+^/acid/alkali: strains (2.0 × 10^8^ cells/mL) were inoculated in TYA medium containing 10 mg/L CuSO_4_, or at acidic (pH 5.0) or alkaline (pH 10.0) conditions, respectively, on a shaker at 37 °C. The *A*_600_ value was measured every 2 h. Tolerance to Cu^2+^/acid/alkali was evaluated according to the growth curve.

### 4.5. Homology Modeling

The amino acid sequences of Ds-26-16 and the variant were submitted to Phyre2 server (available at: http://www.sbg.bio.ic.ac.uk/phyre2). Homology modeling was conducted in intense mode [32], which is based on a profile-profile alignment algorithm. According to heuristics to maximize confidence, percentage identity and alignment coverage, 16 templates (Table S2) were selected for structural modeling.

### 4.6. Cell Surface Scanning with SEM

Strains (1.5 × 10^8^ cells/mL) were inoculated in TYA medium containing 0, 0.51, 1.03 or 1.54 M NaCl for 12 h, respectively, and then were centrifuged at 5000 r/min for 5 min. The precipitate was washed with PBS, fixed with 2.5% glutaraldehyde at 4 °C overnight, dehydrated gradually with 30%, 50%, 70%, 80%, 90%, 100% ethanol (twice) and then dissolved in absolute ethanol. The solution was dropped on the cover glass, dried at room temperature, fixed to the SEM column for spraying gold. The cell surface scanning was carried out with QUANTA 200 type SEM (FEI Company, Hillsboro, OR, USA). A total of 50~100 cells from each sample were randomly selected to measure the cell length.

### 4.7. Antioxidant Enzyme Activity, MDA and NO Content Analysis

Strains (2.2 × 10^8^ cells/mL) were inoculated in TYA medium containing 0, 0.77 or 1.03 M NaCl, respectively. The bacteria were collected by centrifugation at 2, 4, and 8 h, respectively. After ultrasonic cell-break at 4 °C, the supernatant (as crude enzyme solution) was collected by centrifugation at 12,000 r/min for 20 min. CAT and POD activities were determined according to the method described in Liang et al. [33]. One unit of CAT activity is defined as the amount of enzyme that reduces *A*_240_ 0.01 per min. One unit of POD activity is defined as the amount of enzyme that increases *A*_470_ 0.01 per min. MDA content was determined using thiobarbituric acid method according to the method described in Liang et al. [33]. MDA content (μM) = 6.45 × (*A*_532_ − *A*_600_) − 0.56 × *A*_450_. NO content was determined using NO detection kit (Beyotime Biotechnology Company, Shanghai, China) according to the method from the manufacturer. *A*_600_ value of the cell cultures was also determined simultaneously. The enzyme activity and MDA and NO content were calculated according to the number of cells (10^9^ cells).

### 4.8. Statistical Analysis

All the experiments concerning data comparisons were performed three times. Statistical analyses were performed using the S-N-K method of one-way ANOVA or independent-samples *t*-test (95% confidence) with IBM SPSS Statistics 11.0 (SPSS Inc., Chicago, IL, USA). Values with different lower cases are shown as significantly different at *p* < 0.05.

## 5. Conclusions

A random mutagenesis library was constructed using a dITP-mediated EP-PCR method, and an *E*. *coli* expression system was employed for screening. Seven variants including *EP-5* with enhanced salt tolerance were obtained. Homology modeling revealed that the mutation S32P in EP-5 enabled the perpendicular orientation of two bigger α-helixes at the N- and C-terminals changing into a parallel orientation. Expression of *Ds-26-16* and *EP-5* maintained the cellular morphology, increased the intracellular antioxidant capability, and stimulated NO synthesis to confer salt tolerance in *E*. *coli.* This study provides basis for further utilization of Ds-26-16 and the evolutionary gene in salt tolerance breeding.

## Figures and Tables

**Table 1:** The cell length of *E*. *coli* transformants expressing Ds-26-16 or EP-5 under NaCl conditions (μm).

NaCl (M)	pET-21b(+)	Ds-16-16	EP-5
0	2.23 ± 0.61 ^a^	2.37 ± 0.74 ^a^	2.32 ± 0.86 ^a^
0.51	31.25 ± 9.84 ^a^	2.70 ± 0.87 ^b^	2.36 ± 0.72 ^b^
1.03	7.29 ± 6.83 ^a^	4.20 ± 1.72 ^b^	4.77 ± 2.95 ^b^
1.54	1.81 ± 1.22 ^c^	3.06 ± 1.78 ^b^	3.74 ± 2.31 ^a^

The cell length of pET-21b(+), Ds-26-16 and EP-5 was determined after culturing under different salt concentration for 12 h. a, b or c stands for the significant differences (*p* < 0.05) between the strains under the same salt concentration at the same growth time; a is maximal; c is minimal.

## Supplementary Materials

Supplementary materials can be found at www.mdpi.com/1422-0067/17/11/1813/s1.
