# Supplementary material for: Directed Evolution of Dunaliella salina Ds-26-16 and Salt-Tolerant Response in Escherichia coli
Source: Int J Mol Sci. 2016 Oct 29;17(11):1813. doi: 10.3390/ijms17111813 (PMC5133814; doi:10.3390/ijms17111813)
Supplement: Supplementary file 1 [file ijms-17-01813-s001.pdf]

Supplementary Materials: Directed Evolution of *Dunaliella salina Ds-26-16* and Salt-Tolerant Response in *Escherichia coli*

Yuan Guo, Yanping Dong, Xiao Hong, Xiaonan Pang, Defu Chen and Xiwen Chen

Table S1. EP-PCR system for directed evolution of Ds26-16.

PCR Round	Component	Final Concentration
First	TaKaRa 10× PCR buffer (Mg^{2+} free)	1×
	TaKaRa $MgCl_2$	7.5 mM
	$NaNO_2$	1 μM
	TaKaRa Triton X-100	0.01%
	TaKaRa dATP	0.15 mM
	TaKaRa dCTP	0.15 mM
	TaKaRa dTTP	0.1 mM
	TaKaRa dGTP	0.05 mM
	dITP	0.35 mM
	pET-upstream	0.125 μM
	Seq-rew	0.125 μM
	p21-ORF	0.05 ng/μL
	TaKaRa *rTaq* DNA polymerase	0.025 U/μL
Second	TaKaRa 10× PCR buffer (with Mg^{2+})	1×
	TaKaRa dNTPs	0.2 mM
	pET-T7	0.12 μM
	T7-Ter2	0.12 μM
	The product of the first round PCR	0.05 ng/μL
	TaKaRa *rTaq* DNA polymerase	0.02 U/μL

Table S2. Template proteins selected for homology modeling of Ds-26-16.

Template Protein	Library ID	Confidence (%)	% i.d.
ubiquitin-associated protein 1	c4ae4B	19.4	33
retinoblastoma-associated protein	c2azeC	15.7	75
antilipopolysaccharide factor	c2jobA	11.0	100
trypsin inhibitor bwi-2c	c2lqxA	7.9	50
alternative oxidase mitochondrial	c3vvaD	7.9	31
Non-canonical RBD domain	d1ft8a2	7.9	35
DEP domain	d1v3fa	6.3	75
DEP domain	d1w4ma	6.2	50
DEP domain	d2csoa1	6.1	50
capsid protein gamma	c4ftbD	6.0	46
DEP domain	d1uhwa	5.8	50
coat protein gamma	c2z2qF	5.6	46
capsid protein gamma	c4ftbF	5.6	46
cyclin	d2cchb1	5.5	19
capsid protein gamma	c4ftbE	5.5	46
capsid protein gamma	c4fjF	5.5	46

Table S3. Primers used in the study.

Primers	Sequences (5′→3′)
pET Upstream	ATGCGTCCGGCGTAGA
Seq-rew	CGGATATAGTTCCTCCTTTCAGC
pET-T7	GAAATTAATACGACTCACTATAGGGGAA
T7-Ter2	GCTAGTTATTGCTCAGCGGTG
159-EP-F	TGGTGGACAGCAAATGGGTC
159-EP-R	CAGCCGGATCTCAGTGGTGG

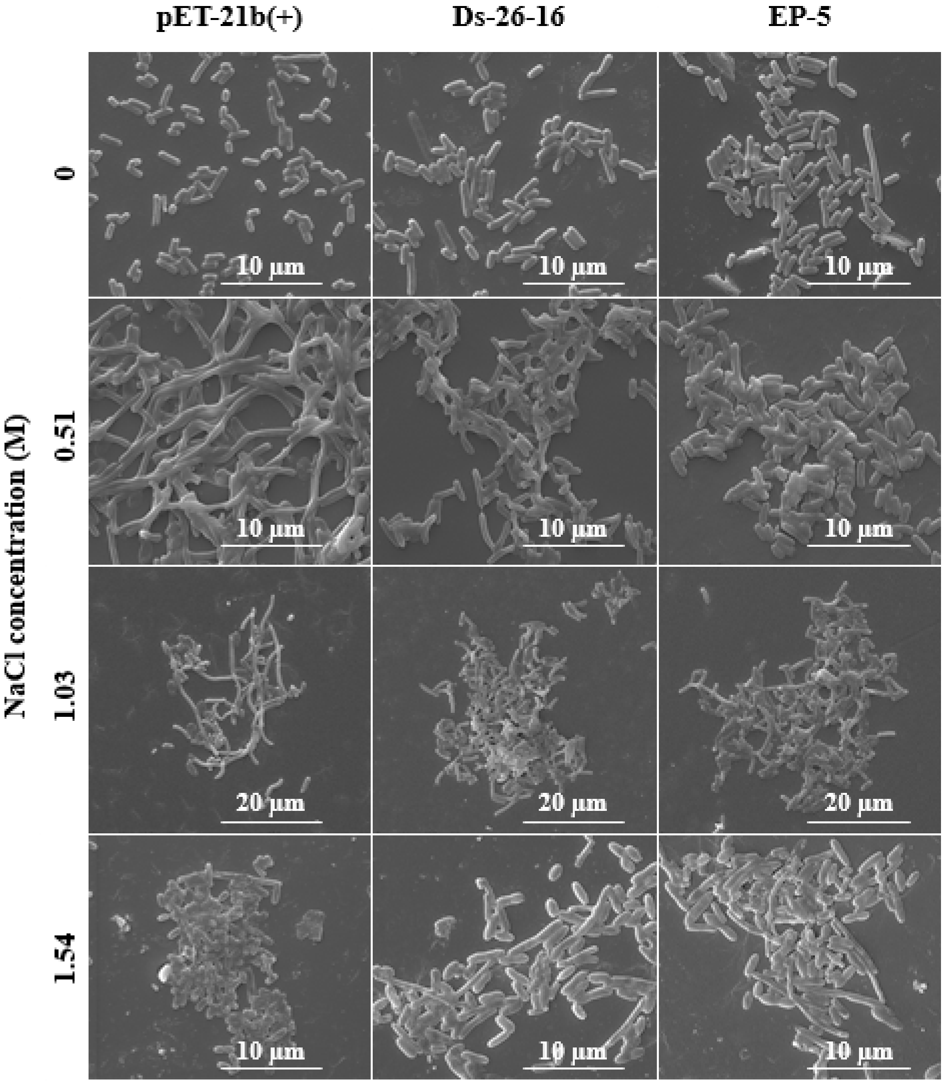

Figure S1. Scanning electron micrograph of *E. coli* transformants expressing Ds-26-16 or EP-5 under different NaCl stress levels. pET-21b(+), Ds-26-16 and EP-5 were cultured under different salt concentrations for 12 h and then the cell length was determined. Det: ETD. HV: 15.0 kV. WD: 9.6 mm.
